# Evaluation of genotype by environment interactions on milk production traits of Holstein cows in southern Brazil

**DOI:** 10.5713/ajas.18.0174

**Published:** 2018-07-26

**Authors:** Raphael Patrick Moreira, Luis Fernando Batista Pinto, Altair Antônio Valloto, Victor Breno Pedrosa

**Affiliations:** 1 Department of Animal Science, Ponta Grossa State University, Ponta Grossa, PR, 84030-900, Brazil; 2 Department of Animal Science, Federal University of Bahia, Salvador, BA, 40170-115, Brazil; 3 Paraná Holstein Breeders Association - APCBRH, Rua Professor Francisco Dranka, Curitiba, PR, 81200-404, Brazil

**Keywords:** Dairy Cattle, Environmental Effects, Genetic Evaluation, Milk Yield, Milk Solids

## Abstract

**Objective:**

This study assessed the possible existence of genotype by environment interactions for milk, fat and protein yields in Holstein cattle raised in one of the most important milk production basins in Brazil.

**Methods:**

Changes in the genetic parameters and breeding values were evaluated for 57,967 animals from three distinct regions of southern Brazil, divided according to differences in climate. The genotype by environment interaction was determined by genetic correlations between regions, estimated by the restricted maximum likelihood, considering the animal model. Bull rankings were investigated to verify the ratio of coincident selected animals between regions for each trait.

**Results:**

The estimates of heritability coefficients were similar between two regions, but were lower in the third evaluated area, for all traits. Genetic correlations between regions were high, ranging from 0.91 to 0.99 for milk, fat and protein yields, representing the absence of a genotype by environment interaction for productive traits. The percentage of selection error between regions for the top 10% of animals ranged from 0.88% to 2.07% for milk yield, 0.99% to 2.46% for fat yield and 0.59% to 3.15% for protein yield.

**Conclusion:**

A slight change in genotype between areas was expected since no significant genotype by environment interactions were identified, facilitating the process of selecting Holstein cattle in southern Brazil.

## INTRODUCTION

Reproductive biotechnology has resulted in the extensive and global distribution of genetic material from animals with high productive potential. For this reason, genotypes that are chosen from areas dissimilar to the region in which they are to be introduced may not satisfy breeder expectations. This aspect may be related to the genotype lacking an adaptation to the territory in which it is introduced; this in turn will affect the genotype by reducing the expression of its maximum productive potential [1]. The genotype×environment interaction (GEI) has several implications that may significantly affect dairy production, including factors responsible for the production of milk, fat and protein. Apart from this, the individual breeding values can be affected, finally culminating in a reclassification of the breeding rankings [2].

In Brazil, a major proportion of milk production comes from the Holstein breed [3]. How ever, very few studies have focused on productive traits in response to the variety of climates prevailing in the different regions of the country. Most temperate countries, particularly the United States, Canada and Netherlands, which have preferred the Holstein breed for a long time, are well-known as big exporters of semen from the breed [4]. They are now expanding their markets, making successful entries into the tropical and subtropical zones of the world, including Brazil. It therefore becomes crucial to assess whether the imported genetic material will meet the performance expected by breeders in various environmental contexts and emphasises the significance of GEI studies in dairy cattle.

The Paraná Basin in southern Brazil is well-known for having the highest dairy productivity per animal in the country. However, it is characterised by sharp climatic diversity, with some regions experiencing temperate climate conditions, while others have subtropical climate [5]. Therefore, such climatic differentiations occurring within the state do exert some influence that can alter the traits of interest to some degree. Furthermore, in Brazil, findings regarding the presence of GEI in the Holstein breed are meagre, with reports being available but limited only in terms of milk production [6] and not fat and protein production. Therefore, the present study focused on assessing the influence of the GEI on the production of milk, fat and protein in Holstein herds in Paraná, Brazil.

## MATERIALS AND METHODS

Data on 305 d milk yield (MY), fat yield (FY), and protein yields (PY) recorded from 57,967 primiparous cows in 375 herds were taken from the database of the Holstein Association in Paraná, Brazil, considering the years between 1990 and 2015. A pedigree file containing the animal’s identification, sire and dam was used, totalling 79,387 animals in the relationship matrix.

The effects included in the model were the fixed effects of contemporary group and the cow age at calving as a covariate (linear and quadratic), beyond the random additive genetic effect. The contemporary groups were created considering the interactions of herd-year-season, with four seasons of the partum being considered, i.e., December to February, March to May, June to August, and September to November. The data were checked and records that included errors, insufficient information, animals of unknown parentage, progenies of bulls which only pertain to one herd, and contemporary groups containing fewer than three animals were removed.

The state of Paraná in southern Brazil is situated between 22° 30′58″ and 26° 43′00″ south latitude and 48° 05′37″ and 54° 37′08″ west longitude and extends across 199,307 km^2^. Based on the climatic classification of the Secretariat of Agriculture of the State of Paraná, Brazil [7], the following different regions were easily distinguished: region 1 (R1): mesothermal climate humid and super humid (lacking a dry season, but experiencing severe winter, severe and frequent frost, rainy and mild summers, at altitudes higher than 850 to 900 m); region 2 (R2): mesothermal climate with no dry season (harsh winter with average rainfall and some frost, rainy summers and high temperature, altitudes normally below 850 to 900 m); region 3 (R3): mesothermal climate with a distinct dry season (hot summers and low occurrence of frost, mostly lower than 850 to 900 m).

Multi-trait analyses were performed using the GEI for a group that included the three regions (R1+R2+R3), for each individual trait, considering the same trait in each region as a distinct characteristic. The connectivity between the herds was assured by connecting sires, maintaining only those that produced at least one cow simultaneously, in at least two of the three regions in the study. A total of 1,016 sires were assessed in this study, genetically connected by region as follows: R1 and R2 (951 sires); R1 and R3 (528 sires); R2 and R3 (480 sires); R1, R2, and R3 (471 sires).

In the general matrix format, the model was represented as given: y = Xb+Za+e, where y is the vector of the analysed trait, b refers to the vector of solutions for the fixed effects containing the contemporary group and covariate of the age at birth, a is the vector of the solutions for the additive genetic random effect, X and Z are the incidence matrices for the fixed and additive genetic effects, respectively, and e is the vector of the random residuals. For the multi-trait analysis, regarding the joint analysis of the different regions, the matrix model was explained as given:

$$\text{Y}=\left[\begin{array}{c} y_{1}\\ y_{2}\\ y_{3} \end{array}\right]=\left[\begin{array}{ccc} X_{1} & 0 & 0\\ 0 & X_{2} & 0\\ 0 & 0 & X_{3} \end{array}\right]\cdot \left[\begin{array}{c} \beta_{1}\\ \beta_{2}\\ \beta_{3} \end{array}\right]+\left[\begin{array}{ccc} Z_{1} & 0 & 0\\ 0 & Z_{2} & 0\\ 0 & 0 & Z_{3} \end{array}\right]\cdot \left[\begin{array}{c} a_{1}\\ a_{2}\\ a_{3} \end{array}\right]+\left[\begin{array}{c} e_{1}\\ e_{2}\\ e_{3} \end{array}\right]$$

where y_1_ is the vector of observations of MY, FY, and PY in R1, y_2_ is the vector of the observations of MY, FY, and PY in R2 and y_3_ is the vector of the observations of MY, FY, and PY in R3, respectively; X_1_, X_2_, and X_3_, are the fixed effects incidence matrices, contained in vectors β_1_, β_2_, and β_3_, respectively; Z_1_, Z_2_, and Z_3_ are the incidence matrices of the additive genetic effects contained in vectors a_1_, a_2_, and a_3_, respectively; e_1_, e_2_, and e_3_ are the random error vectors associated with vectors y_1_, y_2_, and y_3_; 1, 2 and 3 are the R1, R2, and R3 regions, respectively.

$$\left[\begin{array}{c} \alpha \\ e \end{array}\right]\sim NMV\left(\left[\begin{array}{c} 0\\ 0 \end{array}\right],\;\;\;\left[\begin{array}{cc} G & 0\\ 0 & R \end{array}\right]\right)$$

where the G and R matrices are defined as G = G0⨂A and R = R0⨂I, respectively; where G0 refers to the additive genetic covariance matrix of the three regions and R0 is the residual matrix for the three regions where the animal will be observed.

The presence or absence of the GEI was determined by the genetic correlations between the regions, as explained by Falconer [8]. The variance components and genetic parameters were estimated by the restricted maximum likelihood method using VCE6.0 software according to Groeneveld [9], considering the animal model. The breeding values were obtained using PEST software [10]. Additionally, the breeding values and classification of the bulls were submitted to the Pearson and Spearman correlations, respectively, adopting the CORR procedure [11]. Regarding the bulls, the coincident animals between R1, R2, and R3 were established, when 10% of the bulls with the highest predicted transmission ability (PTA) were chosen for each trait in each location, based on the method employed by Pedrosa et al [12].

## RESULTS

The additive, residual and phenotypic variances are shown in Table 1. Table 2 provides the heritabilities for each region and genetic correlations between each region for the three traits. R2 revealed equal heritability for MY in relation to R1. For R1 and R2, the value was 0.21, and for R3 it was 0.16. The same was observed for FY, where R1 and R2 showed similar values, with a heritability value of 0.25 and R3 equal to 0.17. The PY registered less heritability compared with the other traits, with R3, R2, and R1 being 0.10, 0.16, and 0.17, respectively.

The three traits under consideration and among all the regions assessed showed high genetic correlations. For MY, the genetic correlations revealed a value of 0.97 between R2×R3, 0.99 between R1×R3 and 0.93 between R1×R2. In the case of FY the correlations between R2×R3, R1×R3, and R1×R2 were 0.99, 0.94, and 0.93, respectively. PY revealed correlations of 0.93 between R2×R3, 0.99 between R1×R3 and 0.91 between R1×R2. Table 3 shows the Pearson correlations between the regions for the PTA of the bulls for all studied traits. For the three traits between the regions, all the values were confirmed and found to be close to 1. For MY, the Pearson correlation was the highest between the R1×R3 regions (1.00), followed by R2×R3 (0.99), and R1×R2 (0.99). The sequence for FY was R2×R3 (1.00), R1×R3 (0.99), and R1×R2 (0.98). For PY, the correlations showed values of 1.00, 0.99 and 0.98 for R2×R3, R1×R3, and R1×R2, respectively. Among bull ranks, the Spearman correlation also revealed high values. The values for MY were 1.00, 0.99, and 0.99 for R1×R3, R2×R3, and R1×R2, respectively. Relative to FY, the R2×R3 regions registering a correlation of 1.00 showed similarity to R1×R3 with 0.99 and R1×R2 with 0.98. For PY, the R1×R3 value was 1.00, while for R2×R3 it was 0.99 and R1×R2 showed 0.98.

**Figure 1:**
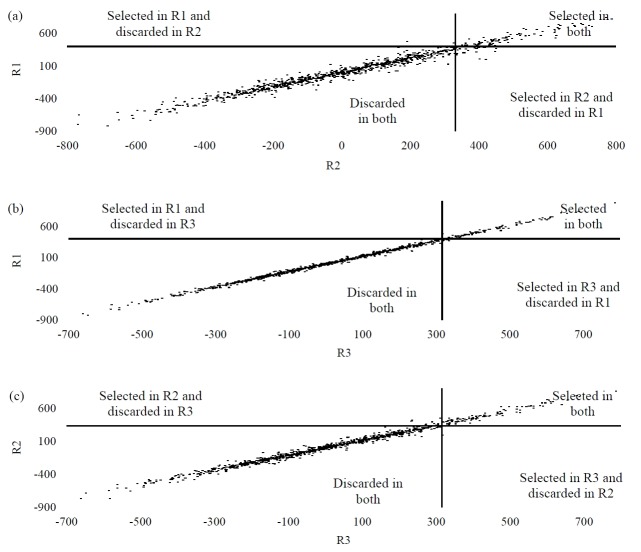
Dispersions of PTAs for milk yield in the quadrants defined by the truncation points of the best 10% bulls for: (a) R1 (PTA ≥385.44) and R2 (PTA ≥363.32); (b) R1 (PTA ≥385.44) and R3 (PTA ≥316.17); (c) R2 (PTA ≥363.32) and R3 (PTA ≥316.17). PTAs, predicted transmission ability.

**Figure 2:**
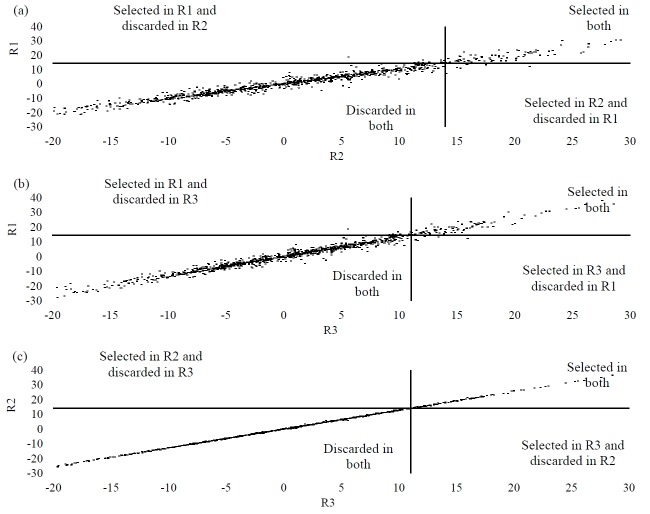
Dispersions of PTAs for fat yield in the quadrants defined by the truncation points of the best 10% bulls for: (a) R1 (PTA ≥14.40) and R2 (PTA ≥13.99); (b) R1 (PTA ≥14.40) and R3 (PTA ≥11.04); (c) R2 (PTA ≥13.99); and R3 (PTA ≥11.04). PTAs, predicted transmission ability.

**Figure 3:**
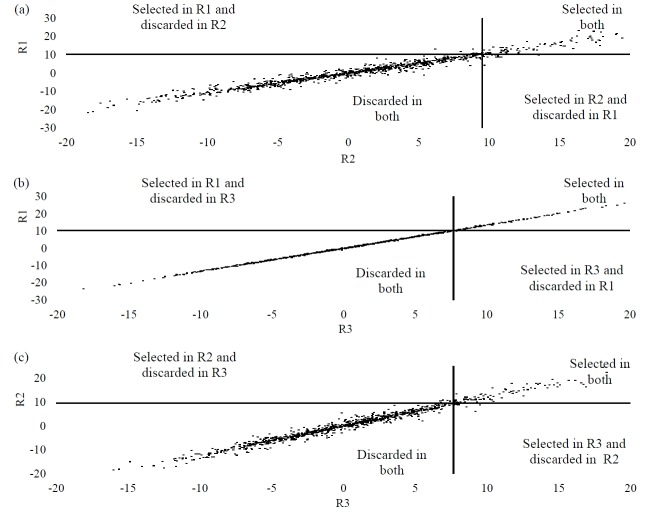
Dispersions of PTAs for protein yield in the quadrants defined by the truncation points of the best 10% bulls for: (a) R1 (PTA ≥10.23) and R2 (PTA ≥9.51); (b) R1 (PTA ≥10.23) and R3 (PTA ≥7.67); and (c) R2 (PTA ≥9.51) and R3 (PTA ≥7.67). PTAs, predicted transmission ability.

Figures 1, 2, and 3 show the PTA dispersions for the three traits in the quadrants established by the truncation points of 10% of the best bulls. Such a dispersion enabled the identification of selection errors in the regions under investigation. For MY, the PTAs that set the truncation points were 385.44, 363.32, and 316.17 for R1, R2, and R3, respectively. The points for FY were 14.40 for R1, 13.99 for R2 and 11.04 for R3. PY revealed the truncation points of the PTAs of the bulls from 10.23 to R1, 9.51 to R2 and 7.67 to R3. Thus, from the PTA dispersions, the proportions of the coincident animals between the regions for the traits under study were assessed. On evaluating the proportion of the coincident animals for MY, values of 98.82% were reported between R2×R3, 99.12% between R1×R3 and 97.93% between R1×R2. For FY, the proportion of the coincident animals was 99.8%, 97.84%, and 97.54% for R2×R3, R1×R3, and R1×R2, respectively. In the case of PY, the proportions recorded were 97.45% for R2×R3, 99.41% for R1×R3 and 96.85% for R1×R2.

## DISCUSSION

From the variances gathered for the three traits in the regions under investigation, it was demonstrated that the environmental variance accounted for a major portion of the total variance in these traits, causing low to moderate heritabilities, especially evident in R3. Greater estimates of the additive genetic variance for the three traits were shown, based on the regions that revealed more productivity, concurring with the findings of Montaldo et al [13]. In this instance, selection increased the additive genetic variance in the herds, and thus raised the estimate of heritability of the traits over the long term.

The heritability values for MY, FY in R1 and R2 were found to be moderate in magnitude and were similar to those reported by Campos et al [14] in their evaluation of data from Holstein cows in Brazilian herds. Huquet et al [15] also found values of moderate to high heritability (from 0.39 to 0.47) for MYs and FYs in their evaluation of Holstein cattle in France. Such high values for heritability recorded in some studies may occur because these herds undergo significant genetic selection in the programs widely adopted in their countries and form adapted genotypes. Additionally, the environmental control practiced in these countries is more intense, utilising more homogeneous production systems and thus minimising the environmental variance. The R3 was the region that presented the lower heritabilities. Apart from this, the heritability results obtained in all three regions for PY concur with the work of Bernabucci et al [16] and Campos et al [14], i.e. 0.15 and 0.17, respectively. The results of 0.17, 0.16, and 0.10 reported for R1, R2, and R3, respectively, reiterate that PY has low heritability. Considering this, it can be concluded that selection for FY has a tendency to reveal greater genetic gains in smaller generations, as compared to PY.

From the genetic correlations applied in the estimation of the presence of the GEI for MY, the changes in the expression of the genotype between R1 and R3 (0.99) because of environmental differences, are less in relation to the comparative among the (R2×R3 = 0.97 and R1×R2 = 0.93). Robertson et al [17] stated that the presence of the GEI is evident when the coefficients of genetic correlations are below 0.80. Therefore, no interaction for MY was evident, because all the regions reported values higher than the one mentioned above. Zwald et al [2] highlighted the role of environment as the possible predominant factor in the effect on production, provided that the manifestations of the genetic components are influenced by the variables such as temperature differences between the regions, for example, characterising the presence of environmental genotype interactions between the different regions. However, the absence of any interaction among the regions reveals that factors including temperature, herd size and influence of genetic material from outside were inadequate to bring about significant alterations in the phenotype expression among the regions being investigated.

The results of the genetic correlations for FY revealed the absence of any significant influence of a GEI as its value fell within the range of 0.93 to 0.99 between the regions. It has already been established that fat is one of the constituents that induces greater instability in milk; variability in FY occurs due to several factors. However, the results reiterate that the changes caused by distinctive genetics among the animals was of no significance. According to the investigation of Montaldo et al [13], the interactions between the FY in Canada, the United States and Chile is influenced by climatic and regional differences, which tend to induce a notable GEI for FY, confirming the reported results.

For PY, variations in the genetic correlations among the evaluated regions were observed from 0.91 between R1 and R2 to 0.99 between R1 and R3. Therefore, it was accepted that R1 appeared to show more similarity in the effects to R3 in terms of the expression of this characteristic, relative to R2. However, from the magnitude of the results shown, the environmental influence was insufficient to induce a significant interaction effect. This occurs because, according to Kolmodin et al [18], alterations in the average rainfall exert little or no effect on the PY, reducing the effects of environmental variance on the total component. However, latitudinal differences have been cited in the literature as the principal reason for the alterations in temperature and time of day [19]. Carabaño et al [20] pointed out that temperature can be a determinant factor that influences the interaction on the production of protein in milk. However, in this study, all the regions of the Paraná were situated within the same latitude range (−30° to −20°). These factors thus reveal that latitudinal differences between the three regions did not exert a noteworthy effect on the response of this characteristic. Finally, this fact can also justify the lack of any remarkable effect on MY and FY, which showed correlations close to 1, as mentioned earlier.

The present study relied on Pearson’s correlations to con firm the veracity of the genetic correlations and further the understanding of the absence of GEI in the Paraná Basin. The results recorded for MY, FY, and PY revealed only very slight distinctions between the genetic values of the animals from one region to another, because the Pearson correlation ranged from 0.98 to 1.00 for the characteristics, between the three regions. Further, the Spearman correlations for the three traits reiterated that the alterations among the bull ranks was low in magnitude between the regions. The values followed Pearson’s standard, ranging from 0.98 to 1.00. Calus and Veerkamp [21] suggested that the GEI might either induce a change in the classifications of the animals, termed reclassification, or reveal only the presence of differences in the breeding values of the animals, without necessitating any classification changes, otherwise termed the scale effect. This emphasises the fact that the animals assessed here showed only slight changes in breeding values between the different regions, with negligible changes in the classification rankings.

Figure 1 shows that the selection error for MY between the regions was in the range of 0.88% to 2.07%, and thus of no significance. This means that the number of bulls selected in one region, when they would be evaluated in another, should not be insignificant. In practice, this confirms the absence of a GEI for MY between the regions, based on the genetic correlations given earlier in Table 2. The best method would be to choose a specific selection program that could include the progenies evaluated in R1 and then the results of PTAs could be extrapolated for the selection of the same animals in the other two regions; then, the percentage of certainty would be around 99.12% for R3 and 97.93% for R2.

This claim was also confirmed for fat and protein production. When considering FY, as revealed in Figure 2, the selection error ranged from 0.99% to 2.46%, and for PY (Figure 3) from 0.59% to 3.15%. Therefore, for these two characteristics, our confidence in a selection program done in R1 and adopted in the other regions would vary from 96.85% to 99.41%, showing up as highly reliable. It is also noteworthy that the regions that exhibited genetic correlations of 0.99, like R1 and R3 for MY, R2 and R3 for FY and R1 and R3 for PY, showed greater uniformity in the bull PTA dispersions.

According to Mulder et al [22], when high genetic correlations are evident between different regions, the highest average genetic gain occurs with only a single selection program, rather than several different programs. The present study supports this assertion because, as previously mentioned, high genetic correlations demonstrate differences of low magnitude in the animal PTAs. Furthermore, as stated above, most of the animals in the Paraná herds were imported from regions experiencing different climatic conditions, which frequently decreased the selection efficiency. Considering these aspects, a cooperative selection program by breeders specifically tailored for such traits would be very acceptable, as it would create animals adapted to these climatic conditions, which could be used by producers in Paraná. It is also the authors’ view that such a methodology, besides increasing genetic progress, could also induce very positive results due to the drop in semen price, thus proving to be advantageous to small producers as well, who form a big part of the primary milk chain in the Paraná Basin.

Based on the findings of this study, it is evident that the three climatic regions assessed in the Paraná Basin in Brazil showed no significant GEI for the traits assessed in the Holstein breed. The breeding values of the animals and their classifications revealed no alterations, irrespective of the regions where they were produced. This makes it clear that the genetic predictions concluded in these regions assessed can, without significant bias, be utilised between regions. Additionally, regarding the absence of any influence exerted by the GEI, a genetic selection program alone will be required for the Holstein dairy herd in Paraná, indicating heightened efficiency and lowered additional expenditure for the implementation of development techniques.

## Figures and Tables

**Table 1:** Additive genetic variance ($\sigma_{a}^{2}$), residual variance ($\sigma_{e}^{2}$) and phenotypic variance ($\sigma_{p}^{2}$) for milk yield (MY), fat yield (FY), and protein yield (PY) for Region 1, Region 2, and Region 3

Items		$\sigma_{a}^{2}$	$\sigma_{e}^{2}$	$\sigma_{p}^{2}$
MY	Region 1	555,716.51	2,043,254.33	2,598,970.84
	Region 2	504,443.32	1,929,089.12	2,433,532.44
	Region 3	368,608.08	1,952,364.42	2,320,972.50
FY	Region 1	786.91	2,386.74	3,173.65
	Region 2	792.87	2,345.70	3,138.56
	Region 3	480.05	2,363.86	2,843.91
PY	Region 1	380.47	1,850.11	2,230.58
	Region 2	363.63	1,838.68	2,202.31
	Region 3	211.02	1,872.70	2,083.71

**Table 2:** Heritability (diagonal) and genetic correlation (above diagonal) for milk yield (MY), fat yield (FY), and protein yield (PY) in Region 1, Region 2, and Region 3

Items		R1	R2	R3
MY	Region 1	0.21	0.93	0.99
	Region 2	-	0.21	0.97
	Region 3	-	-	0.16
FY	Region 1	0.25	0.93	0.94
	Region 2	-	0.25	0.99
	Region 3	-	-	0.17
PY	Region 1	0.17	0.91	0.99
	Region 2	-	0.16	0.93
	Region 3	-	-	0.10

**Table 3:** Pearson correlations (above diagonal) and Spearman correlations (below diagonal) for milk yield (MY), fat yield (FY) and protein yield (PY) in Region 1, Region 2, and Region 3

Items		R1	R2	R3
MY	Region 1	-	0.99	1.00
	Region 2	0.99	-	0.99
	Region 3	1.00	0.99	-
FY	Region 1	-	0.98	0.99
	Region 2	0.98	-	1.00
	Region 3	0.99	1.00	-
PY	Region 1	-	0.98	1.00
	Region 2	0.98	-	0.99
	Region 3	1.00	0.99	-
